# Cellulose production increases sorghum colonization and the pathogenic potential of *Herbaspirillum rubrisubalbicans M1*

**DOI:** 10.1038/s41598-019-40600-y

**Published:** 2019-03-11

**Authors:** Thalita Regina Tuleski, Valter Antônio de Baura, Lucélia Donatti, Fabio de Oliveira Pedrosa, Emanuel Maltempi de Souza, Rose Adele Monteiro

**Affiliations:** 1 Department of Biochemistry and Molecular Biology, Federal University of Parana, Curitiba, Paraná Brazil; 2 Department of Cellular and Molecular Biology, Federal University of Parana, Curitiba, Paraná Brazil

## Abstract

Three species of the β-Proteobacterial genus *Herbaspirillum* are able to fix nitrogen in endophytic associations with such important agricultural crops as maize, rice, sorghum, sugar-cane and wheat. In addition, *Herbaspirillum rubrisubalbicans* causes the mottled-stripe disease in susceptible sugar-cane cultivars as well as the red-stripe disease in some sorghum cultivars. The xylem of these cultivars exhibited a massive colonisation of mucus-producing bacteria leading to blocking the vessels. A cluster of eight genes (*bcs*) are involved in cellulose synthesis in *Herbaspirillum rubrisubalbicans*. Mutation of *bcsZ*, that encodes a 1,4-endoglucanase, impaired the exopolysaccharide production, the ability to form early biofilm and colonize sorghum when compared to the wild-type strain M1. This mutation also impaired the ability of *Herbaspirillum rubrisubalbicans* M1 to cause the red-stripe disease in *Sorghum bicolor*. We show cellulose synthesis is involved in the biofilm formation and as a consequence significantly modulates bacterial-plant interactions, indicating the importance of cellulose biosynthesis in this process.

## Introduction

Nitrogen fixation, solubilisation of phosphorus, production of phyto-hormones and siderophores are some of the mechanisms that plant growth promoting rhizobacteria (PGPR) use to modulate plant growth^1^. Among the described species of *Herbaspirillum*, only *H. frisingense, H. seropedicae* and *H. rubrisubalbicans* are diazotrophic^2^. In addition to fixing nitrogen, *H. rubrisubalbicans* is capable of colonising inner tissues and promote growth of rice, wheat and sugar-cane^3,4^. Interactions between *H. rubrisubalbicans* and roots begin with attachment of the bacteria to the root-surface followed by colonisation of the points of emergence of secondary roots or through cracks in the epidermis. Invasion and endophytic establishment in intercellular spaces, xylem vessels and parenchyma cells then occurs^5–8^.

*Herbaspirillum rubrisubalbicans* is a pathogen of the sugar-cane B-4362 as well as of some sorghum cultivars on which it provokes mottled-stripe and red-stripe diseases, respectively^9,10^. Massive colonisation of the meta- and proto-xylem by mucus-producing bacteria leads to blocking the vessels, decreased photosynthesis and consequently necrosis results^5,6^.

The bacterial mucus can be composed of several substances, including exopolysaccharides. Cellulose is an important component of bacterial exopolysaccharides (EPS) and is known to play a role in the interactions of other associative organisms such as *Rhizobium* with legumes^11,12^.

The cellulose biosynthesis genes are listed under different names in various bacteria including *bcs* (bacterial cellulose synthesis), *acs* (*Acetobacter* cellulose synthesis), *cel* (cellulose) or *wss* (wrinkly spreader). Mutation in these genes in *Pseudomonas fluorescens*, *P. syringae* and *Gluconacetobacter xylinus* (formerly *Acetobacter xylinus*) affected, in fact, cellulose production^13–15^. The operon *bcsABCZ* of *H. rubrisubalbicans* M1 encodes to enzymes for cellulose biosynthesis^7^. *bcsA* and *bcsB* genes encodes two catalytic subunits of cellulose synthase^16^, *bcsC* encodes a periplasmic protein that is involved in cellulose synthesis, *bcsZ* encodes a endo-β-1,4-glucanase (cellulase)^17^ being involved in the cellulose production regulation^18^. Cellulases play essential roles in cellulose biosynthesis; mutation of *bcsZ* in *G. xylinus* resulted in decreased cellulose production and irregular packing of cellulose fibrils^19^. A *bcsZ R. leguminosarum* mutant strain produced longer cellulose microfibrils but the ability to form biofilms was reduced^20^.

A *H. rubrisubalbicans bcsZ* mutant (originally called *wssD*) was impaired in attachment to maize roots when compared with the wild-type strain^7^. In *Rhizobium leguminosarum* and *Agrobacterium tumefaciens* cellulose seems to be important to anchor the bacteria to the roots and promote an efficient and strong attachment^21,22^.

Cellulose is known as component of the biofilms of *G. xylinum*^23^, *Escherichia coli*, *Salmonella enteriditis*, *S. typhimurium*^24,25^ and *P. fluorescens*^13^. Biofilms promote bacterial survival in various environments especially by facilitating horizontal gene transfer as well as integrating the metabolism of the biofilm inhabitants^26,27^. Added to this, the biofilms also confer important advantages to pathogens especially by facilitating colonisation of the host and increasing the resistance to anti-microbials. *Pseudomonas aeruginosa* produces biofilms around *Arapidopsis thaliana* roots, killing the plant in a few days^28^. A mutant of *Salmonella enteritidis* in the *bcs* operon (*bcsC* and *bcsE* genes) produces a fragile, unstable and easily removed biofilm^29^.

In this work the role of the *bcs* genes of *H. rubrisubalbicans* in cellulose production, biofilm formation, colonisation of sorghum roots and leaves, and pathogenicity was further investigated.

## Results and Discussion

### Production of Cellulose by *H. rubrisubalbicans* Strains

**Figure 1:**
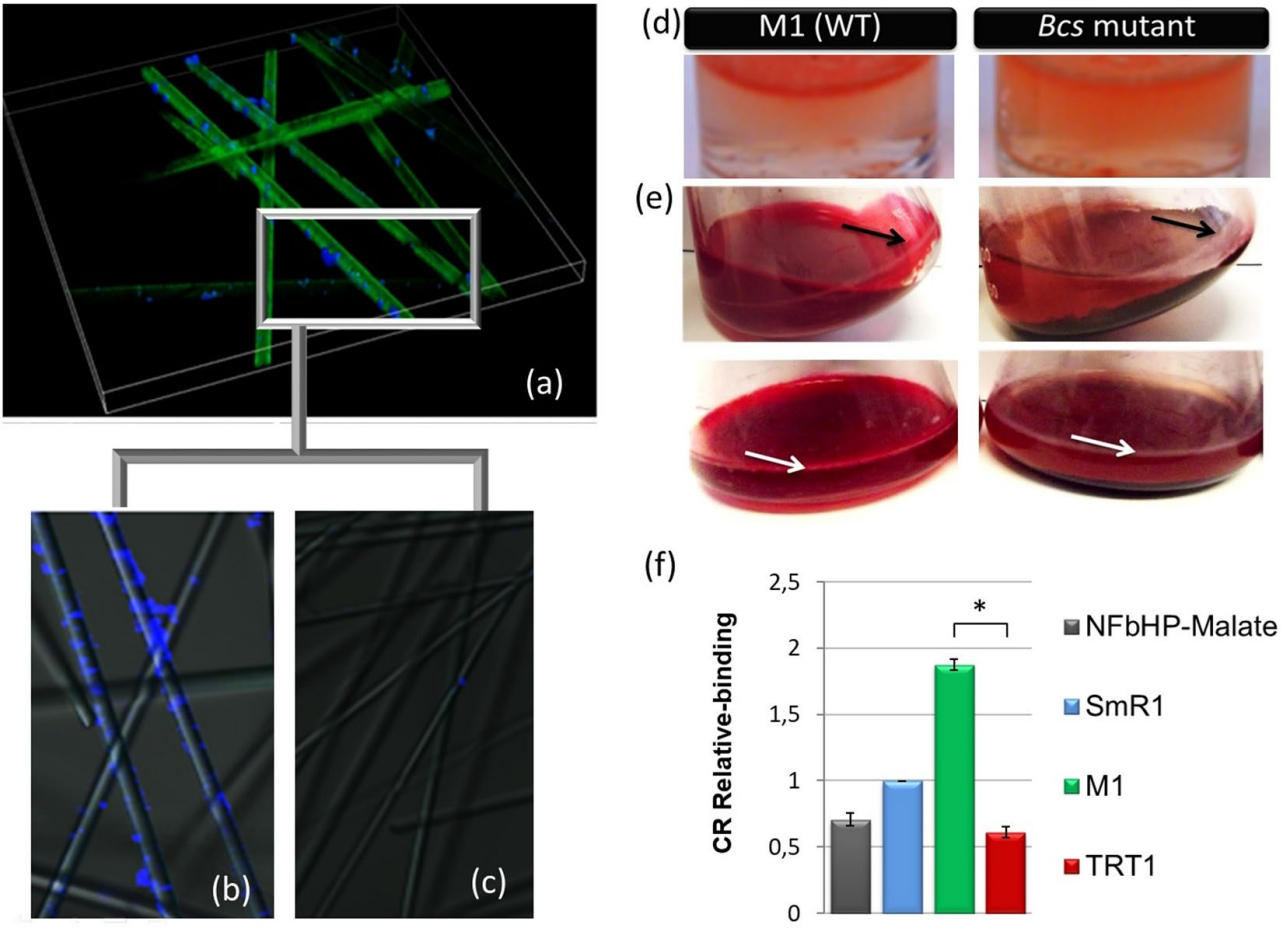
Confocal microscopy of biofilm in inert surface. The wild-type M1 was grown for 18 hours in liquid media in the presence of glass fibres. (**a**) Shows a 3D image of the biofilm structure stained with calcofluor. (**b**) After 18 hours inoculation the bacteria were incubate with buffer for 2 hours at 37 °C, stained with calcofluor and observed by confocal microscopy. In (**c**), biofilm of wild type M1 on glass fibres was incubated with cellulase (0.2 U) for 2 hours at 37 °C, stained and observed by confocal microscopy. The strains M1, and TRT1 (*bcsZ*) were grown in semi-solid (**d**) and liquid (**e**) NFbHPN medium containing 0.005% of congo-red (CR) for 2 days. Biofilm formed in the liquid surface (white arrows) represents a biofilm immersed in a matrix of bacterial exopolysaccharides. Black arrows indicate biofilm strongly attached to the glass. In the *bcs* mutants little or no biofilm adhered to the glass wall was observed. In (**f**), graphs of Relative binding to the CR (congo-red). The CR relative binding was determined by measuring the bound-CR/OD_600_. The controls used were only the NFbHPN medium with the dye congo-red. Relative CR-binding is calculated relative to *H. seropedicae* SmR1 – a non cellulose producer. The SmR1 strain was considered with a relative binding of one (1) to normalize the data.

To confirm the presence of cellulose structures in the *H. rubrisubalbicans* M1 bacterial biofilm, we perform a microscopy assay during the biofilm formation in glass fibres. The bacteria were grown in media containing the fibres until visible biofilms formed (18–24 hours). Then, the cells were stained with calcofluor a fluorescent dye (excitation wavelength-360 nm)^30^ that binds β(1-4)-linked glucans. In these experiments biofilm formation started with *H. rubrisubalbicans* M1 aggregating on the glass fibres eventually forming clumps that were intensively stained by calcofluor (Fig. 1a). To confirm that the stained structures contained cellulose, the bacteria was grown for 18 hours and treated with mock solution or cellulase. After 2 hours the cells were observed by confocal microscopy. In Fig. 1b the fibrils are extensively stained by calcofluor. The brightly stained cellulose-calcofluor complex disappeared after treatment with cellulase (Fig. 1c), indicating that this stained-structures are, in fact, cellulose.

To better determine the importance of the *bcs* operon, the gene *bcsZ* (coding to 1,4-endoglucanase) was inactivated by insertion, producing strain TRT1 (*bcsZ* mutant strain).

The cellulose production by the wild-type and mutant strains was confirmed by congo-red staining. The wild-type and TRT1 (*bcsZ*) *H. rubrisubalbicans* strains were grown on semi-solid NFbHPN medium containing congo-red. The wild-type M1 strain was strongly stained when compared with TRT1 (Fig. 1d). A similar result was observed in cells adhered to the walls of the flask containing cultures in liquid medium (Fig. 1e). The results suggest difference in cellulose production by the analysed strains.

Given that the amount of congo-red bound to the bacterial cells is proportional to the amount of cellulose attached to them, the congo-red relative binding was determined and the results show that the strain M1 produced more than twice as much cellulose as the TRT1 (*bcsZ*) strain (Fig. 1f), indicating that the *bcsZ* mutant strain are defective in cellulose production. *H. seropedicae* SmR1, a no cellulose producing strain, had approximately the same congo-red relative binding as the control flask with only NFbHPN medium. Mutant strains of *Rhizobium leguminosarum* bv. *trifolii* (*celA*, *celB*, *celE*, *celR2* genes) impaired in cellulose production also showed a reduction in congo-red binding^21^.

The rate of sedimentation of bacteria is related to the amounts of EPS on the bacterial surface^31^. Sedimentation of TRT1 (*bcsZ*) (3% of sedimentation per hour) was slower than that of the wild-type strain M1 (10% of sedimentation per hour) (data not shown). After 6 hours, the wild-type had 60% of sedimentation, while TRT1 strain had 18.1% and 16.9%, respectively (data not shown), suggesting that the size of bacterial aggregates was smaller than those produced by strain M1 under these growth conditions.

The *H. rubrisubalbicans bcs* operon includes a gene for cellulase involved in the correct formation of fibrils. To confirm the impairment of the whole operon, we also performed a cellulase assay using CM cellulose as substrate to determine if the cellulolitic activity of the mutant strain was impaired by *bcsZ* disruption. Figure S1 shows that the TRT1 strain have a lower cellulase activity, confirming that disruption of the *bcsZ* genes caused reduction of 1,4-endoglucanase activity.

The results indicate the mutation in *bcsZ* gene led to a severe perturbation in cellulose biosynthesis and a reduction in cellulose degradation in *H. rubrisubalbicans*. Similar results were described for *K. xylinus* where *bcsZ* is necessary for cellulose biosynthesis and proper fibrila packing^16^.

### Cellulose and Biofilm Formation on Glass Fibres

**Figure 2:**
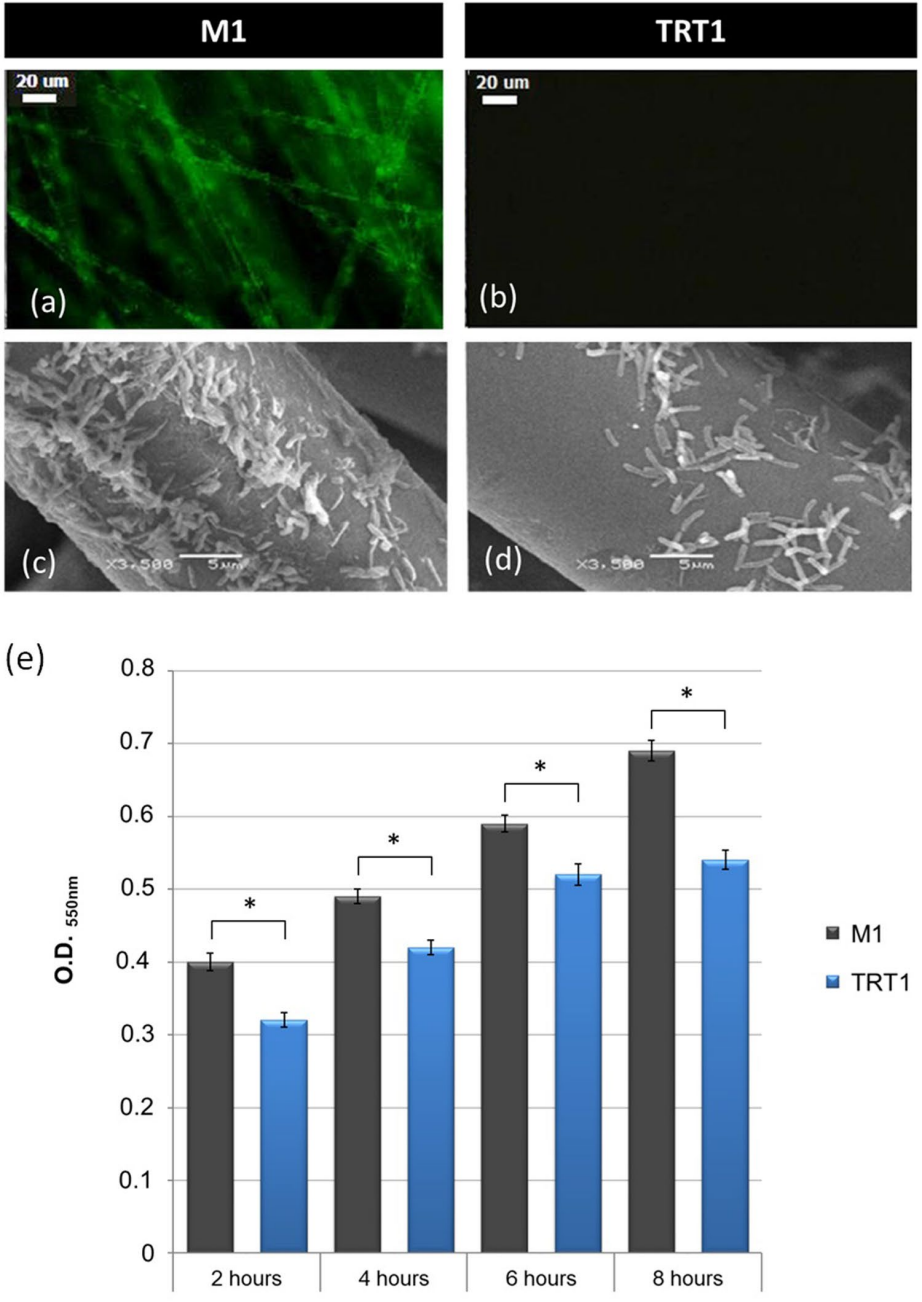
Biofilm formation on inert matrix. (**a**,**b**) Biofilm formation on glass fibers after 24 hours of inoculation observed by fluorescence microscopy after calcofluor staining. (**c**,**d**) *H. rubrisubalbicans* strains grown in liquid media containing glass fibres 8 hours after inoculation observed by SEM. Magnifications and scales are shown in the micrographs. (**e**) Biofilm quantification after crystal=violet staining in the indicated times. The results represent the average of three independent assays performed in duplicate. (*) Significantly different at p ≤ 0.01 (Student t test, Assistat program).

The role of cellulose in adhesion of *H. rubrisubalbicans* M1 to inert surfaces was tested using glass fibres. Cellulose in the biofilm matrices was also revealed by staining with calcofluor. After 24 hours incubation, stained product was only evident in glass fibres incubated with the wild-type M1 (Fig. 2a). Binding of calcofluor was not observed in fibres incubated with the *bcs* mutants, because of the lower amounts of the biofilm attached to the fibres (Fig. 2b).

Knowing that cellulose is involved in biofilm development we analysed the biofilm by SEM (Scanning Electron Microscope) (Fig. 2c,d) and number of cells attached to the glass fibres by crystal violet staining (Fig. 2e). In agreement with calcofluor staining, the biofilm of TRT1 (*bcsZ*) strain attached to the fibres was significantly lower than that of the wild-type strain throughout the experiment.

The scanning electron-microscopy (SEM) performed in the same condition showed significant differences in early biofilm formation (8 hours growth), confirming that cellulose is important for the formation of bacterial biofilms (Fig. 2c,d). The micrographs also show that the bacteria attached to the root surface are better organised and more compact in the wild-type strain M1 as compared to those of the *bcs* mutant (Fig. 2c,d).

**Figure 3:**
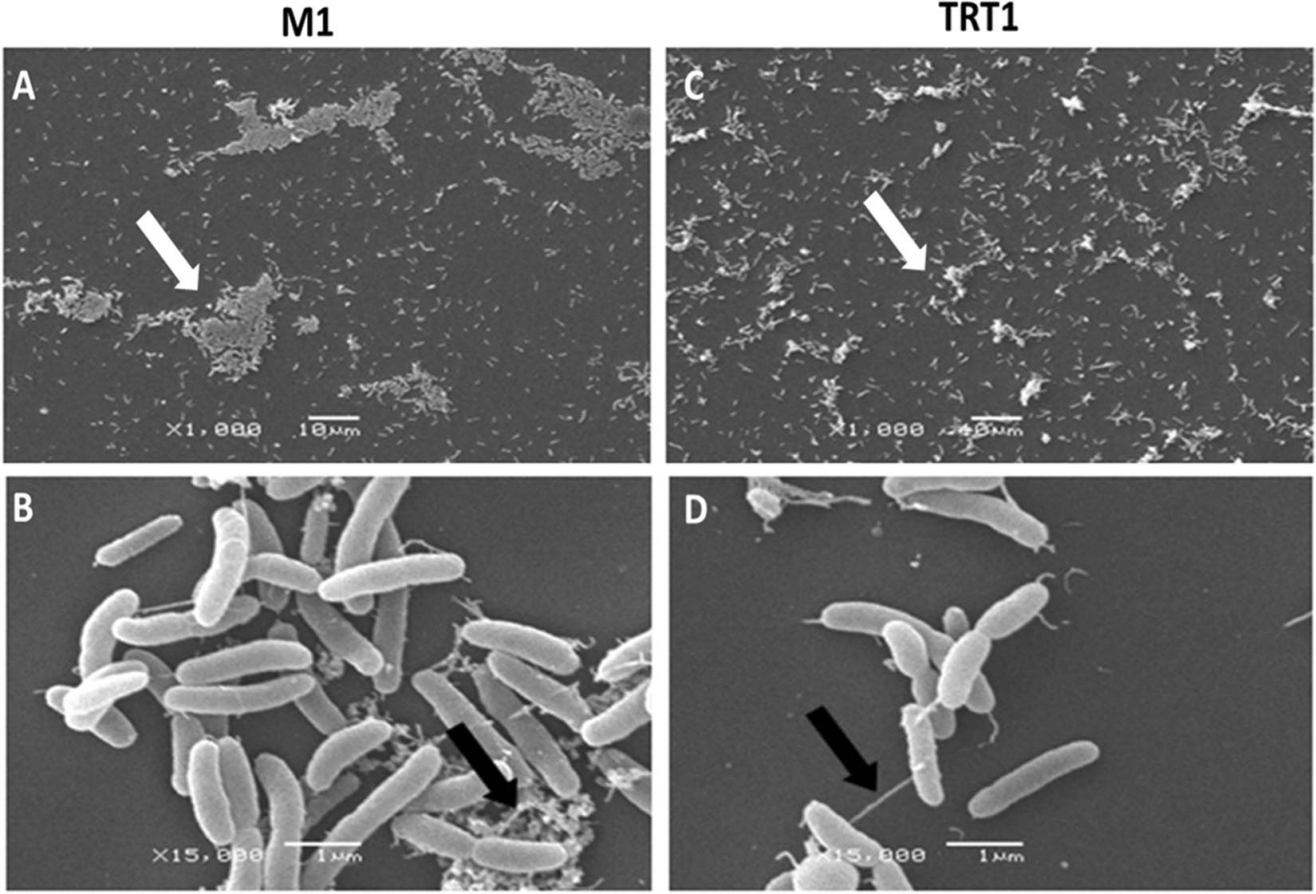
Scanning Electron Microscopy of *H.rubrisubalbicans* strains M1 and TRT1. The bacteria were grown in liquid medium containing cover glass with 1 cm of diameter on the bottom. The cover glass recovered after 72 hours were observed and the images show bacterial biofilm formed. The increased use and scales are shown in micrographs. (**a**,**b**) Showed the M1 strain. (**c**,**d**) Showed the TRT1 strain. The white arrows indicate the bacterial aggregates. The black arrows indicate the difference in the arrangement of bacterial fibrils observed in the aggregates.

*Herbaspirillum rubrisubalbicans* also formed biofilms on cover glass in static liquid cultures. After 72 hours incubation, the biofilms attached to the cover slips were analysed by SEM. Biofilms formed by strain TRT1 were fragile and easily removed in contrast to those formed by strain M1 which were more compact and adhered to the cover glass more strongly (Fig. 3a,d). The M1 aggregates were larger and more tightly packed than those of the TRT1 mutant. Fibrila characteristic of polysaccharides were prominent in biofilms formed by the wild-type strain (Fig. 3b). *Rhizobium sp*. mutant in *celC2* gene had longer cellulose microfibrils and presented drastic reduction in biofilm formation^20^.

The results show that mutation in *bcsZ* gene of *H. rubrisubalbicans* led to reduction in biofilm formation, probably as a result of impaired cellulose production and changes in fibril organization.

Mutation in another gene of the operon, the *bcsA* gene coding to a cellulose synthase subunit (strain TRT2), led to similar phenotypes observed in strain TRT1 confirming that the *bcs* operon is important for cellulose biosynthesis and biofilm formation in *H. rubrisubalbicans* M1 (Figs S2 and S3).

### Influence of cellulose on motility of *H. rubrisubalbicans*

Mutation in *bcsZ* gene also affected motility of *H. rubrisubalbicans*. Comparison of the *bcsZ* mutant growing on semi-solid medium with the wild-type strain, showed that the former halo spread more slowly, forming a halo 44% smaller (data not shown) when compared with the wild-type strain, suggesting that reduced content of cellulose also alters motility of *H. rubrisubalbicans*.

### Cellulose and *H. rubrisubalbicans* interactions with plants

Previous work had shown that the numbers of the cellulose-deficient mutant bacteria that attached to maize roots was 53-fold lower than the wild-type^7^. Since *H. rubrisubalbicans* M1 can be pathogenic to some plant cultivars, we used maize and sorghum to test the effect of mutation in *bcs* genes on colonisation. *H. rubrisubalbicans* M1 is capable to colonize maize roots both endophytically and epiphytically without producing any disease symptom. On the other hand, sorghum (BR007A) is susceptible to the *H. rubrisubalbicans* M1 and upon inoculation will develop the red-stripe disease.

**Figure 4:**
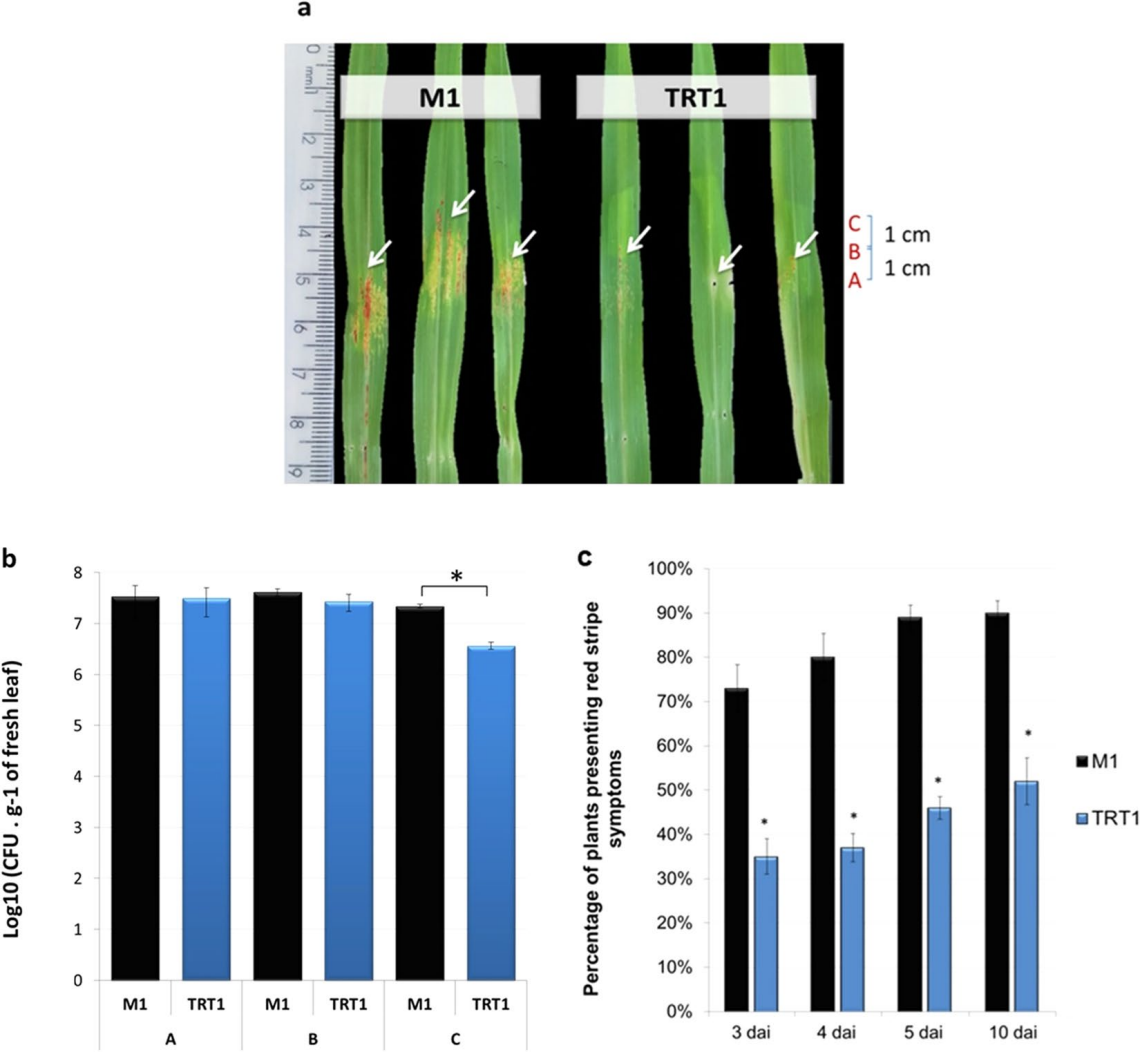
(**a**) Sorghum symptoms of red-stripe disease after 7 days of inoculation with M1 and *bcs* mutant strain. Arrows indicate the regions with disease symptoms. (**b**) Number of bacteria colonizing leaves of sorghum 7 days after inoculation. The points A, B and C are indicated in the panel (a). (**c**) Percentage of sick plants after inoculation with 10^6^ cells of M1 and TRT1 on the stalk. Significant difference between M1 and TRT1 with a significance level of p ≤ 0.05 (*) and p ≤ 0.01 (**) (t test, Assistat program).

To analyse if the mutation of the cellulose cluster affected the ability of *H. rubrisubalbicans* to promote the red-stripe disease in sorghum, 10^6^ cells of the strains M1, and TRT1 were inoculated with a hypodermic needle in the stalk of 10 days old sorghum plants. Seven days after inoculation the symptoms of the red-stripe disease were observed in the leaves (Fig. 4a) and the number of bacteria colonizing leaf tissues were determined (Fig. 4b). The number of bacteria present 3 cm from the inoculation point (Point C indicated in Fig. 4a) was 6 times smaller in the TRT1 mutant in comparison with the wild-type M1. Also, the percentage of plants presenting the red-stripe disease symptoms 3, 4, 5 and 10 days after inoculation with M1 were much higher when compared to the mutant TRT1 (~90% compared with 50%) (Fig. 4c), suggesting that cellulose plays a role on the development of the red-stripe disease.

To analyse if the colonisation would be altered in susceptible and non-susceptible plants we did the colonization assay in resistant maize and a susceptible sorghum.

**Figure 5:**
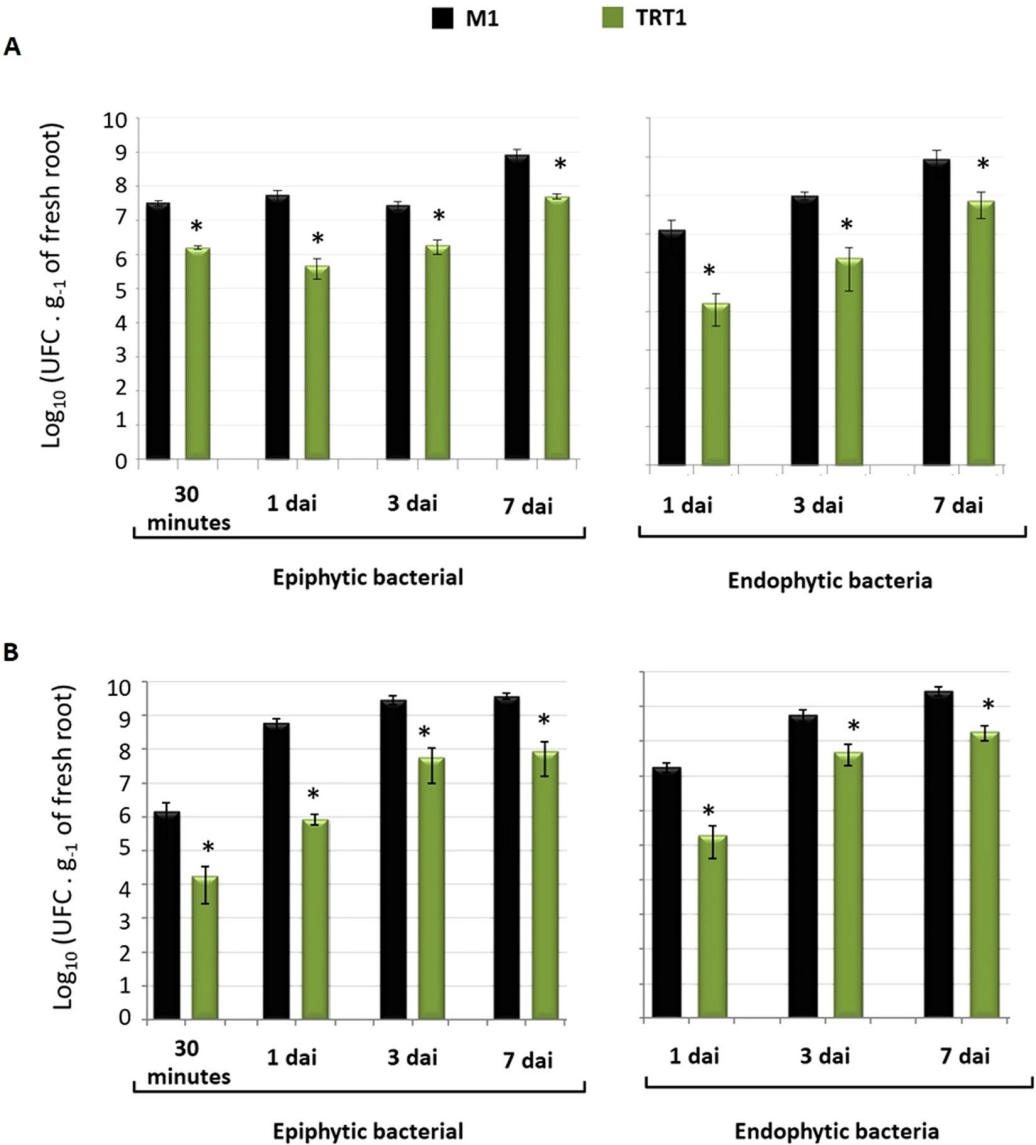
Sorghum (**a**) and Maize (**b**) root colonization by *H. rubrisubalbicans* M1 and TRT1 strains. The numbers of epiphytic and endophytic cells were determined 30 minutes, 1,3 and 7 days after inoculation (d.a.i.). The data represent the average of at least 3 biological replicates with 5 experimental determinations each. (*) Significant statistical differences were observed between M1 and TRT1 with a significance level of p > 0:05. Significant difference between M1 and TRT1 with a significance level of p ≤ 0.05 (*) (t test, Assistat program).

Figures 5a and b show the epiphytic sorghum and maize roots colonization, respectively. In all analysed days the colonization was significantly lower (10–100 fold) in TRT1 mutant when compared with the wild-type M1.

SEM studies of ephyphitic colonisation of maize roots 1, 3 and 7 days after inoculation with *H. rubrisulbalbicans* strains M1 and TRT1 are shown in Fig. S4 (a–c - wild-type and d–f - TRT1). One day after inoculation (d.a.i.) single cells and discrete bacterial agregates associated with the periclinal cell wall surface can be seen in seedlings inoculated with the both strains (Fig. S4a,d). Aggregates of the wild-type strain cells are clearly observed at 3 dai (Fig. S4b), while only single cells of mutant strain are observed colonizing the plant root (Fig. S4e). At day 7 still large number of cells and agregates were seen in the wild type strain (Fig. S4c). In contrast, biofilm and aggregate formation was severly restricted in the mutant colonised roots (Fig. S4f). Also, a treatment of *H. rubrisubalbicans* with cellulase before inoculation of maize led to 10-fold decrease in bacterial attachment of the wild-type strain to root-cells (from 3 × 10^6^ to 5 × 10^5^ CFU).

The mutation in *bcsA* (namely TRT2) gene caused the same phenotype observed for the strain containing a mutation in *bcsZ* gene, reduced virulence in sorghum, and a decrease in endophytic and epiphytic colonization in sorghum and maize roots (Figs S5 and S6).

We also carried out maize growth promotion experiments. The wild type strain stimulated maize root growth seven days after inoculation. The mutant strain TRT1 showed similar results, except for root volume which was significantly smaller than those colonized by the wild-type strain (Fig. S7). These results suggest that the *bcsZ* gene is not essential for plant growth promotion in maize.

Together the data suggest that cellulose production by *H. rubrisubalbicans* is not essential but it is involved in epiphytic and endophytic colonisation both in beneficial or pathogenic interaction. Recently, Mitra and coworkers^32^ reached similar conclusions in their study of rice root colonization by LPS-deficient *Rhizobium*. The results indicate that competent epiphytic colonization of root by rhizobacteria is an important trait for subsequent endophytic colonization.

In summary, the results show that cellulose synthesis is important for cell aggregation, biofilm formation, colonisation of gramineae roots and also for development of the red-stripe disease in sorghum by *H. rubrisubalbicans*.

## Methods

### Strains, plasmids and growth conditions

The strains and plasmids used at this work are described in Table 1. *H. seropedicae* was used as a negative control in cellulose production experiments.

**Table 1:** Strains and plasmids.

Strains and Plasmids	Relevant Characteristic	Source or Reference
***H. rubrisubalbicans***		
M1	Wild Type	Baldani *et al*.^8^
TRT1	Km^R^, *bcsZ*^*-*^	Monteiro *et al*.^7^
TRT2	Km^R^, at *bcsA*^*-*^	This work
***H. seropedicae***		
SmR1	Z78 but SmR 100 μg/mL, Nif+	Pedrosa *et al*. 2001
*E. coli*		
Top10	*hsdR, mcrA*, *lacZΔM15, recA*	INVITROGEN
Plasmids		
pTZ57R/T	AmpR, TA cloning vector	FERMENTAS
pHRTRT2	pTZ57R/T, with *bcsA* fragment	This work

The *H. seropedicae* SMR1, *H. rubrisubalbicans* M1 and its mutant strains TRT1 and TRT2 were grown in NFbHPN (with malate as carbon source) medium^33^ under agitation (120 rpm), 30 °C for 18 to 24 hours. Semi-solid and solid NFbHPN media contained 0.175 and 1.5% (w/v) agar, respectively. Kanamycin at a final concentration of 500 μg.mL^−1^ was added to media used with the cellulose-deficient mutants TRT1 and TRT2.

The *E coli* strain was grown in LB medium^34^ at 37 °C and 180 rpm, with appropriated antibiotics.

### *bcsA* mutagenesis

The plasmid pHRTRT2 contains an internal fragment of the *bcsA* coding region and a cassette that confers resistance to kanamycin (Km). This plasmid was electro-transformed in *H. rubrisubalbicans* M1 and a mutant strain, named TRT2, was selected by kanamycin resistance. Insertion of the recombinant plasmid into the genome of the mutant strain was confirmed by PCR.

### Determination of cellulase activity in *H. rubrisubalbicans*

Cellulase actvity was determined according to Kasana (2008)^35^. The strains were spot inoculated in NFbHPN plates containing 0.5% carboxymethylcellulose (CMC). The cultures were grown for 1 to 6 days at 30 °C. The plates were stained with iodine solution (0.6% KI and 0.33% I_2_) and the extent of CMC digestion was measured as the halo diameter.

### Effect of cellulase on *H. rubrisubalbicans* attachment to maize roots

Strain M1 was grown to an O.D._600_ of 1.0, treated with 250 µg.mL^−1^ or 0.2 U of cellulase (Sigma Aldrich - 22178) for two hours at 30 °C and washed three times with sterile saline. Treated bacteria were used to inoculate two days old maize seedlings (10^5^ bacteria/seedling). After 30 minutes exposure, the seedlings were washed three times with sterile saline and bacteria attached to the roots recovered by vortexing for 45 seconds in sterile saline. Then, the seedlings were removed and the bacteria present in the supernatant counted by serial dilution.

### Motility assays

Aliquots (20 µL) of bacterial suspensions (10^8^ bacteria.mL^−1^) were spot inoculated in NFbHPN medium containing 0.25% agar in Petri dishes. The cultures were incubated statically at 30 °C and the diameter of the halo was determined 48 hours later.

### Sedimentation rate

The bacterial strains were grown in liquid NFbHPN medium (30 °C, shaken at 120 rpm to an O.D._600_ = 1). Then 1 mL of cultures was kept static and the superficial supernatant sampled after 10, 20, 40, 60, 180 and 360 minutes. CFU were determined by serially diluting the samples and plated on solid NFbHPN.

### Biofilm formation on glass fibres

Biofilm formation was carried out in liquid NFbHPN medium incubated with 50 mg glass fibres. Cultures were incubated at 30 °C, 120 rpm. After incubation, one sample was prepared for scanning electron microscopy (SEM) as described above and in another equal sample the glass fibres were recovered, washed with saline and stained with 200 µL of crystal violet for 5 minutes. Then glass fibres were washed five times with saline to remove non-bound crystal-violet and de-stained with 1 mL 70% (v/v) ethanol. Absorbance of ethanol solution at 550 nm was used as an index of biofilm formation.

### Biofilm formation on cover slips

Bacteria were grown in Petri dished containing liquid NFbHPN medium and 1 cm diameter glass cover-slips. After 72 hours of static growth at 30 °C, the cover-slips were recovered, fixed and prepared for SEM as described above.

### Scanning electron microscopy

Both cover glass, glass fibres and biological samples were fixed in Karnovsky solution [2% (w/v) paraformaldehyde, 2.5% (w/v) glutaraldehyde in 0.1 M cacodylate buffer pH 7.2 at 4 °C)^36^, dehydrated in ascending alcohol and acetone series, and the critical point attained in a Bal-Tec CPD - 030 Critical Point Dryer (CEPSR, New York, USA) using carbon dioxide. Plating with gold was performed in a Balzers SCD – 030 Sputter Coater (Oerlikon Balzers, Liechtenstein). SEM analyses were performed in a JEOL-JSM 6360 LV (Akishima, Tokyo, Japan) scanning electron microscope.

### Staining of *H. rubrisubalbicans* cells with congo-red

After two days growth, 0.005% (w/v) congo-red solution was added to liquid or semi-solid NFbHPN static cultures of *Herbaspirillum* spp. The resulting stained pellicle formed in the air-liquid interface was recovered, vortexed, centrifuged and the optical density of supernatant was determined spectrophotometrically at 550 nm. The amount of dye bound was calculated by the difference between *H. seropedicae* strain SmR1, a non-cellulose producer, and *H. rubrisubalbicans* cultures. Since SmR1 lacks all cellulose synthesis genes and does not produce cellulose^7^ it was used as reference for comparisons with *H. rubrisubalbicans*.

### Staining of *H. rubrisubalbicans* with calcofluor

*H. rubrisubalbicans* strains were grown in liquid NFbHPN medium (30 °C, 120 rpm, 24 hours) containing 50 mg of glass fibres. Glass fibres was then recovered, stained with 50 mM calcofluor for 1 hour, washed with saline and observed under a confocal microscope.

To determine the role of cellulose, biofilms were first treated with cellulase (250 ug.mL^−1^ or 0.2 U.mL^−1^), then stained with calcofluor and observed under a confocal microscope. Confocal images were obtained using a Nikon Ti Microscope (Nikon Corp, Tokyo, Japan). The images were scanned using an AxioCam camera (Carl Zeiss Microscopy GmbH, Jena, Germany) attached to the microscope. AxioVision LE software v. 4.6 (Carl Zeiss) was used to the analyse the images. Tri-dimensional images were generated using Nikon’s NIS-Elements software.

### Plant colonisation assays

Seeds of two maize cultivars (SHS 3031 and SHS 5050) and sorghum (BR007A) were disinfected for 20 minutes in 1% (w/v) sodium hypochlorite and 0.4% (v/v) Tween 20, then washed with 70% (v/v) ethanol for five minutes followed by four washes with sterile water. The seeds were then germinated on 1% agar-water plates for 48 to 72 hours at 28 to 30 °C in the dark. The seedlings were inoculated with bacterial suspensions containing 10^5^ bacteria.mL^−1^. Then, the seedlings were transferred to glass tubes containing 20 mL of plant medium^37^ and 20 g polypropylene spheres and kept at 25 °C under light (12 hours photoperiod).

For determination of epiphytic root colonisation, the numbers of attached bacteria were determined by recovering the roots from the tubes, washing them three times with saline, vortexed for 1 minute, then the supernatant was serially diluting and plating onto solid NFbHPN medium.

For determination of endophytic root colonisation, the roots were recovered and surface disinfected with 1 minute washes with 1% (w/v) sodium hypochlorite, 70% (v/v) ethanol and sterile dH_2_0. After sanitization the roots were macerate, serially diluted and, plated onto solid NFbHPN medium.

For determination of leaves colonisation, a hole punch 0.5–0.7 cm in diameter was recovery for the leaf on the indicated regions. The samples were washed 3 times and crushed with 1 mL of saline solution (NaCl 0.9%). The suspension was serial diluted and plated in NFbHPN.

For the leaf colonisation assays, the leaves were recovered after the stipulated times, washed 3 times and macerated with saline solution (NaCl 0.9%). The suspension was serial diluted and plated in NFbHPN. The point A is the centre point of the symptom development. The point B is 1 cm above the point A and the point C is 2 cm above the point A.

The plates were kept in growth chamber at 30 °C.

For plant growth experiments, maize seeds disinfection, germination and inoculation were as before. Seven days after inoculation, plants were harvested, the roots scanned with the Epson Expression LA2400 scanner and the data processed with the WinRHIZO pro software (Regent Instruments, http://www.regentinstruments.com/).

### Pathogenicity assays

Seeds of sorghum (BR007A) were sterilised as described above. The seeds were then germinated on 1% agar-water plates for 48 to 72 hours at 28 to 30 °C in the dark. After germination the plants were transferred to a pot containing sterile vermiculite as substrate. The plants were kept at 25 °C under light (12 hours photoperiod). Seven days after the planting, the plants were inoculated with a hypodermic needle with 10^6^ bacterial cells 2 cm above the substrate. The plants were observed during 7–10 days for symptom development.

## Supplementary information

Supplementary Information
