# Supplementary material for: Cellulose production increases sorghum colonization and the pathogenic potential of Herbaspirillum rubrisubalbicans M1
Source: Sci Rep. 2019 Mar 11;9:4041. doi: 10.1038/s41598-019-40600-y (PMC6412066; doi:10.1038/s41598-019-40600-y)
Supplement: Supplementary file 1 — Supplementary Information [file 41598_2019_40600_MOESM1_ESM.pdf]

Cellulose production increases sorghum colonization and the pathogenic potential of *Herbaspirillum rubrisubalbicans* M1

Thalita Regina Tuleski[1], Valter Antônio de Baura[1], Lucélia Donatti[2], Fabio de Oliveira Pedrosa[1], Emanuel Maltempi de Souza[1], Rose Adele Monteiro[1*]

[1]Department of Biochemistry and Molecular Biology, Federal University of Parana, Curitiba, Paraná, Brazil.

[2] Department of Cellular and Molecular Biology, Federal University of Parana, Curitiba, Paraná, Brazil.

*Correspondence to Rose Adele Monteiro <roseadele@gmail.com>
Postal address: Centro Politécnico, Setor de Ciências Biológicas, Jardim das Américas, Postal Box: 19046, Curitiba, PR – Brazil
Telephone: +55 (41) 33611667
Fax: +55 (41) 32622042

Supplemetary information

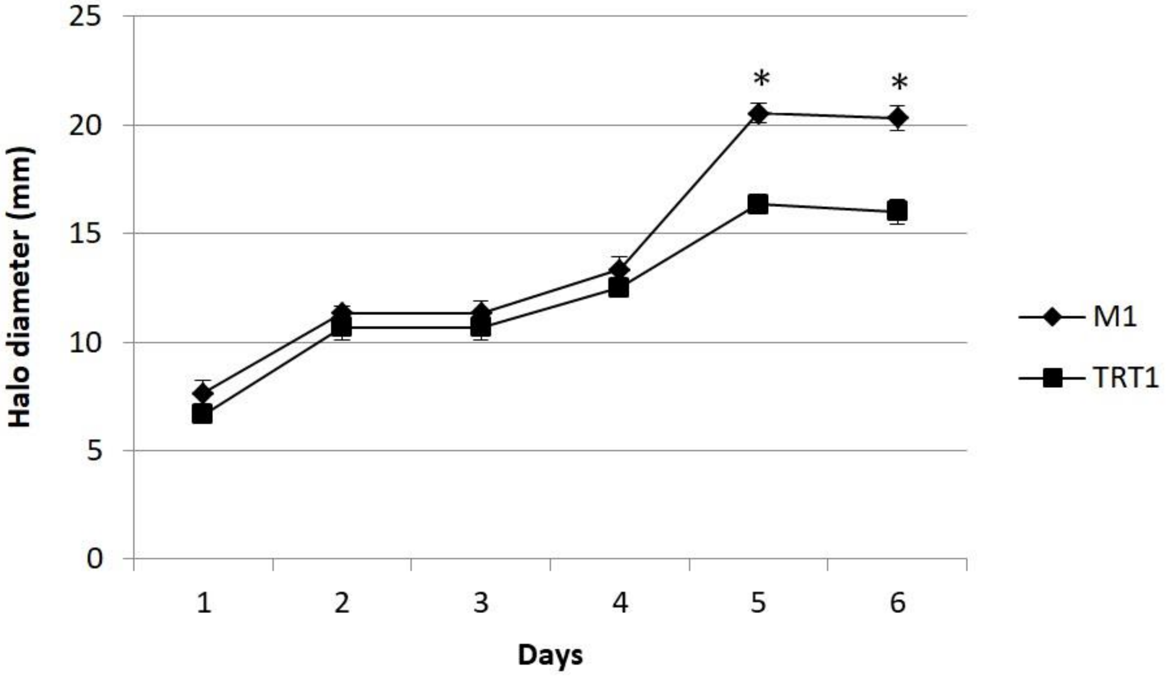

Fig. S1 - Cellulase activity of wild-type and the TRT1 strains. 10^6 bacteria were inoculated in NFbHPN plates containing 0.5% of CMC (carboximetilcellulose). After the 1, 2, 3, 4, 5 and 6 days the plates were stained with iodine solution for evaluation of the enzymatic activity halo. The results represent average of 3 experiments performed in duplicate and (*) means significant differences with significance level of $p<0.01$ between analyzed points (student t test, Assistat program).

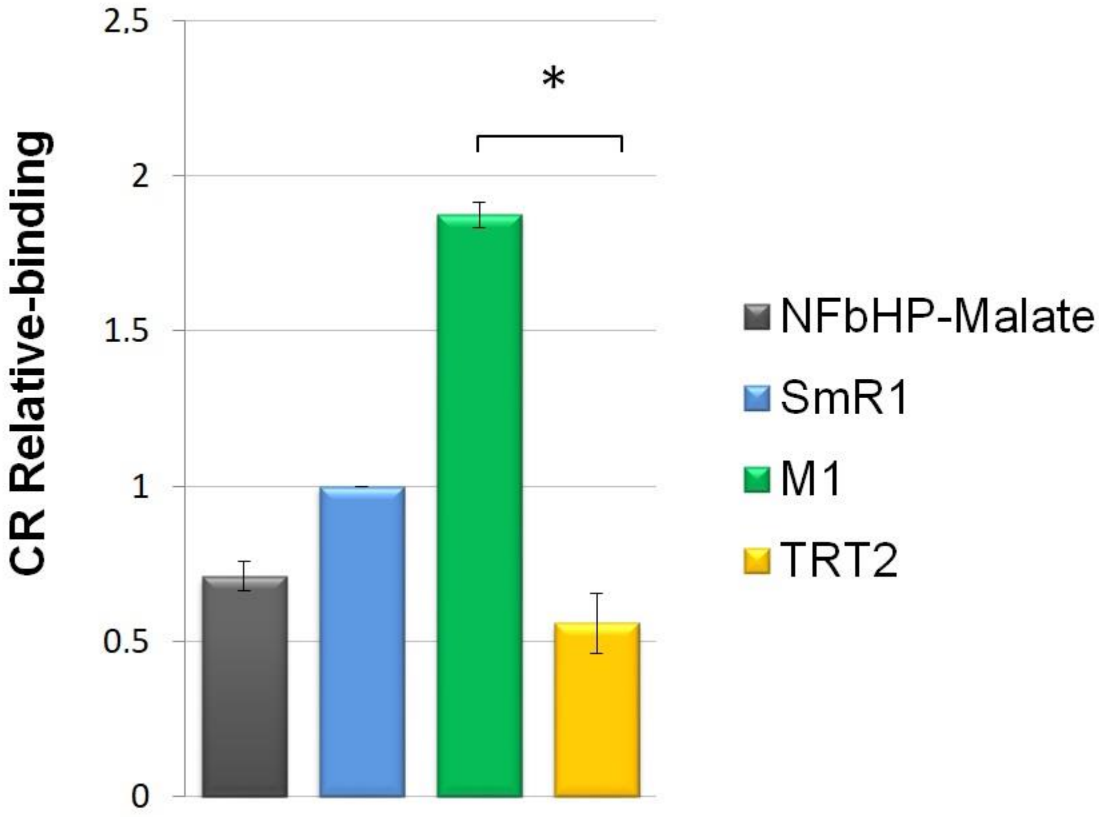

Fig. S2 – M1 and TRT2 relative binding to the CR (congo-red). The CR relative binding was determined by measuring the bound-CR/OD_{600}. The controls used were only the NFbHPN medium with the dye congo-red. Relative CR-binding is calculated relative to *H. seropedicae* SmR1 – a non cellulose producer. The SmR1 strain was considered with a relative binding of one (1) to normalize the data.

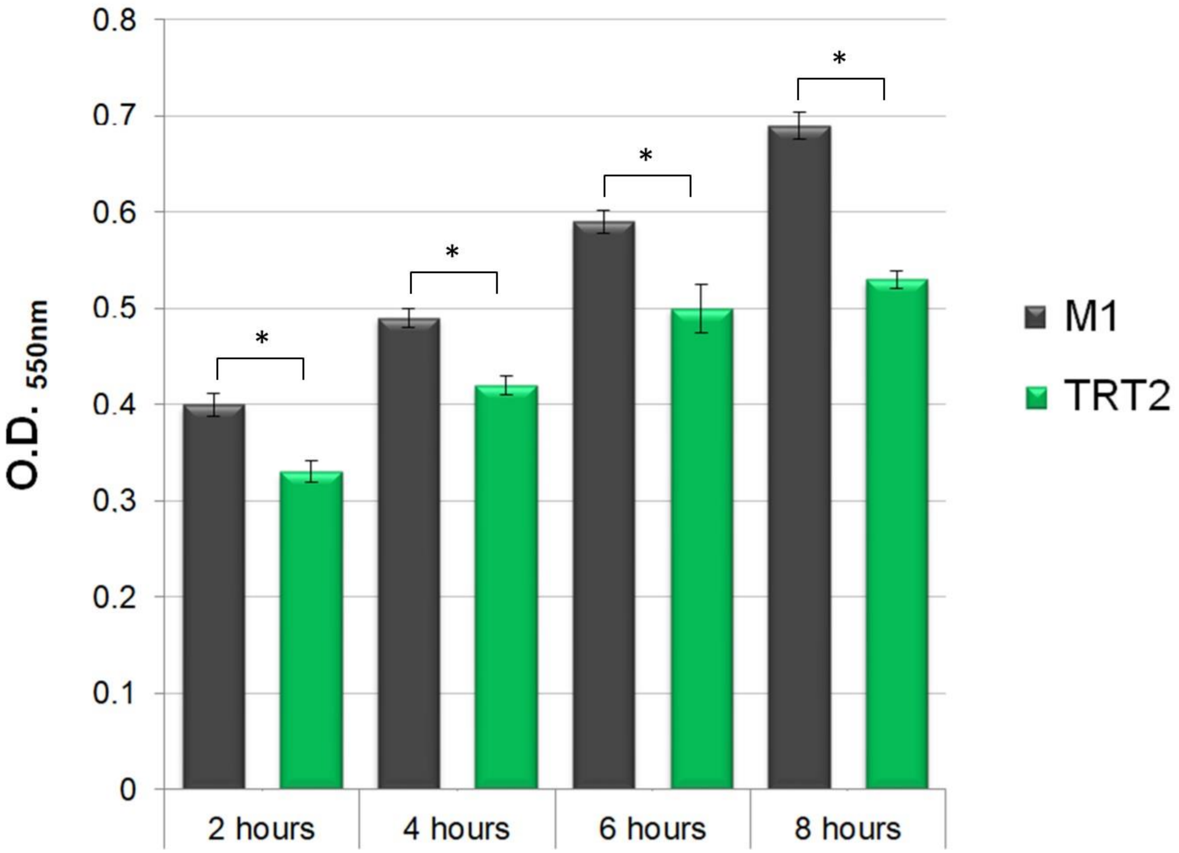

Fig. S3 – M1 and TRT2 biofilm quantification after crystal violet staining in the indicated times. The results represent the average of three independent assays performed in duplicate. (*) Significantly different at p≤0.01 (Student t test, Assistat program).

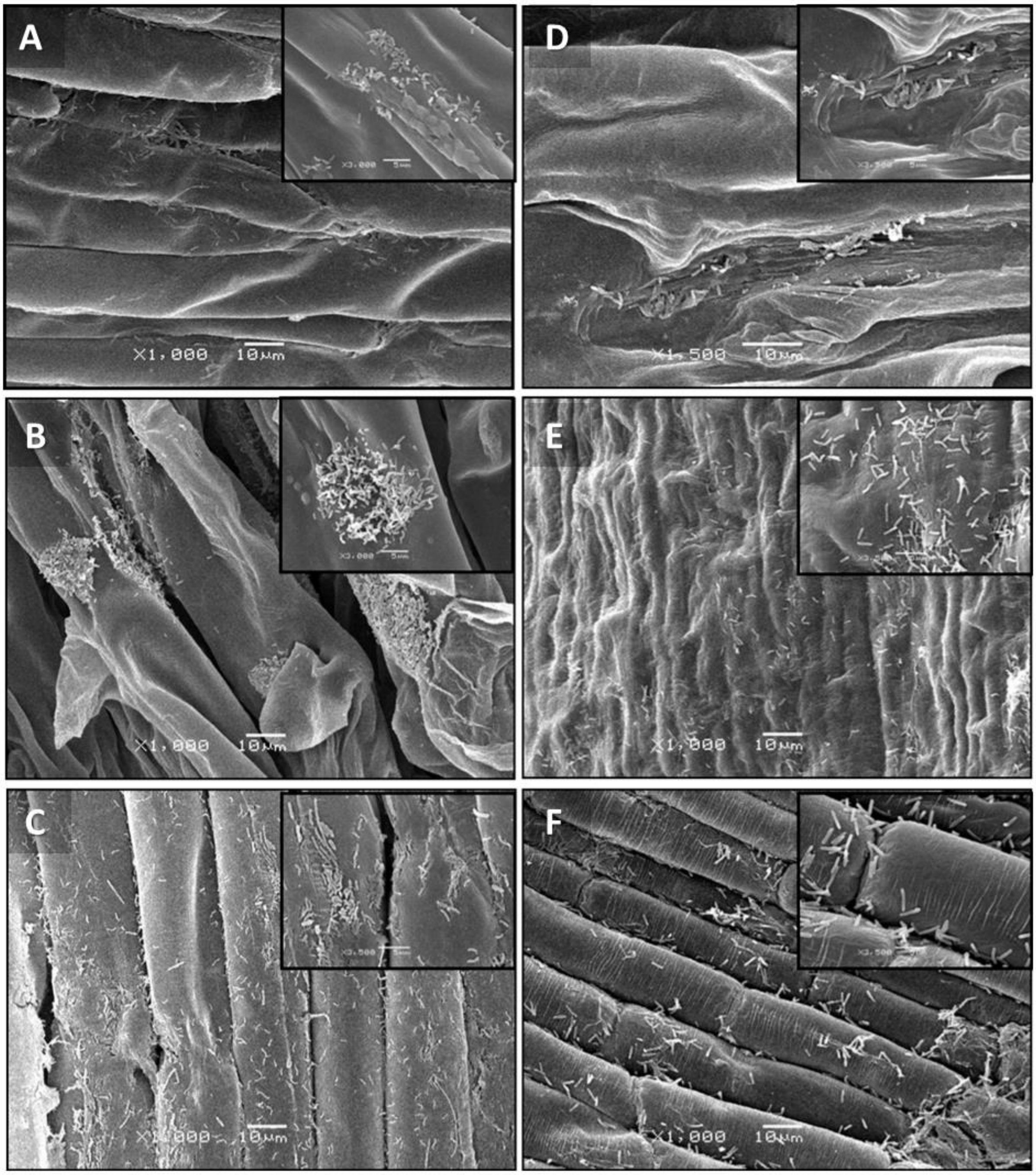

Fig. S4 – Epiphytic maize colonization by *H. rubrisulbalbicans* M1 and TRT1. Maize seedlings were inoculated with 10^5 bacteria and grown in plant medium. After 1, 3 and 7 days, the roots were prepared and analysed by SEM. A, B and C show the colonization of the wild type strain M1 after 1, 3 and 7 days, respectively. D, E and F show the colonization of the mutant strain TRT1 after 1, 3 and 7 days of inoculation, respectively. Magnification and scales are shown in the images.

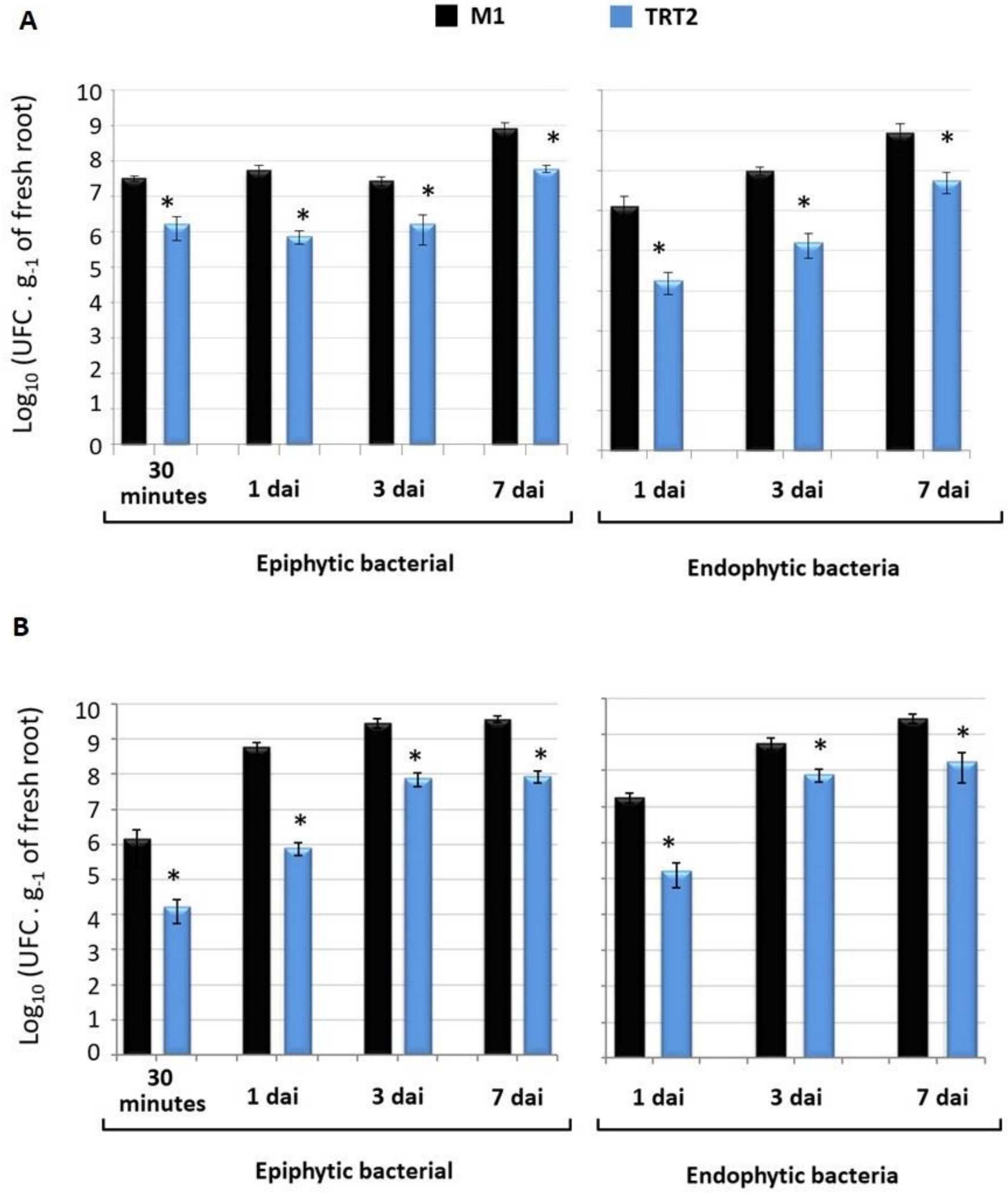

Fig. S5 - Sorghum (a) and Maize (b) root colonization by *H. rubrisubalbicans* M1and TRT2 strains. The numbers of epiphytic and endophytic cells were determined 30 minutes, 1,3 and 7 days after inoculation (d.a.i.). The data represent the average of at least 3 biological replicates with 5 experimental determinations each. (*) Significant statistical differences were observed between M1 and TRT2 with a significance level of $p > 0:05$. Significant difference between M1 and TRT2 with a significance level of $p \leq 0.05$(*) (t test, Assistat program).

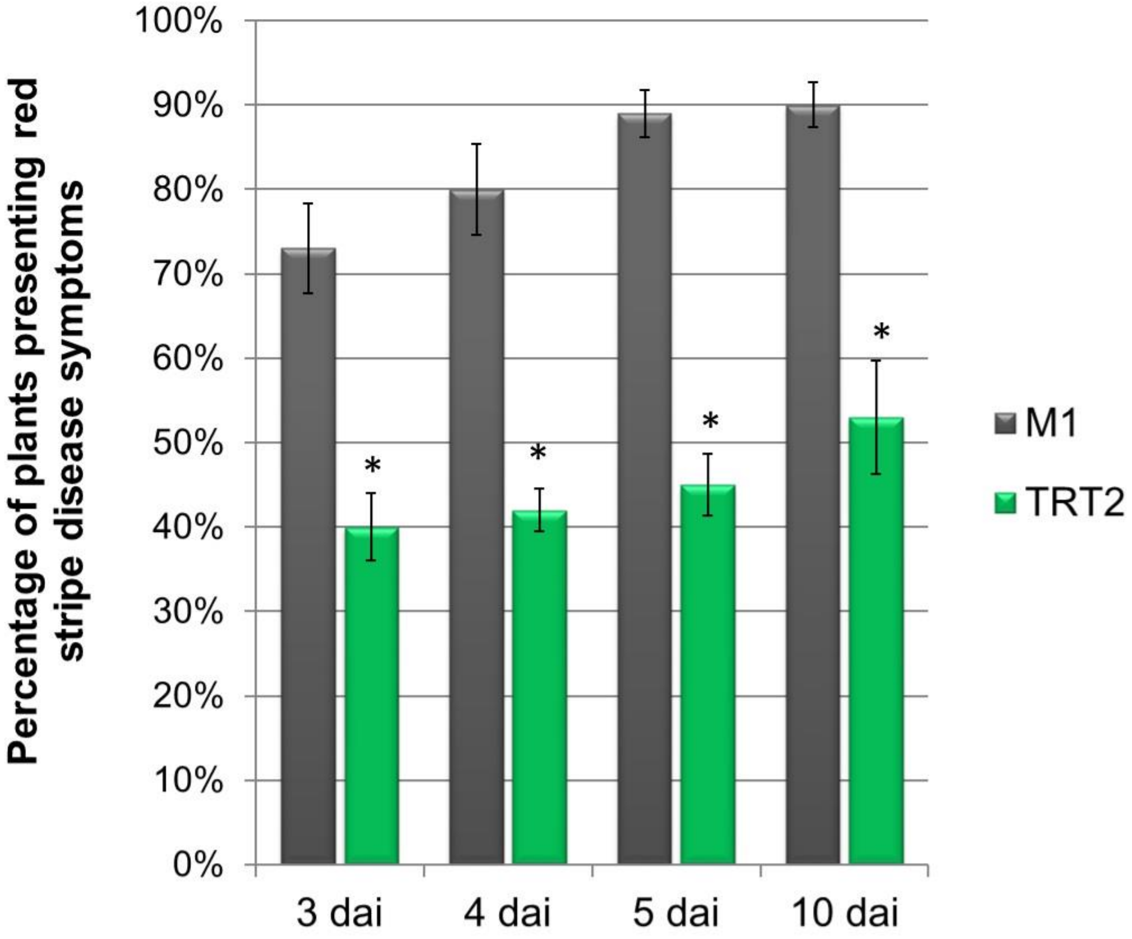

Fig. S6 - Percentage of plants with red stripe disease symptoms after inoculation with 10^6 cells of M1 and TRT2 on the stalk. Significant difference between M1 and TRT2 with a significance level of $p \leq 0.05$(*) and $p \leq 0.01$ (**) (t test, Assistat program).

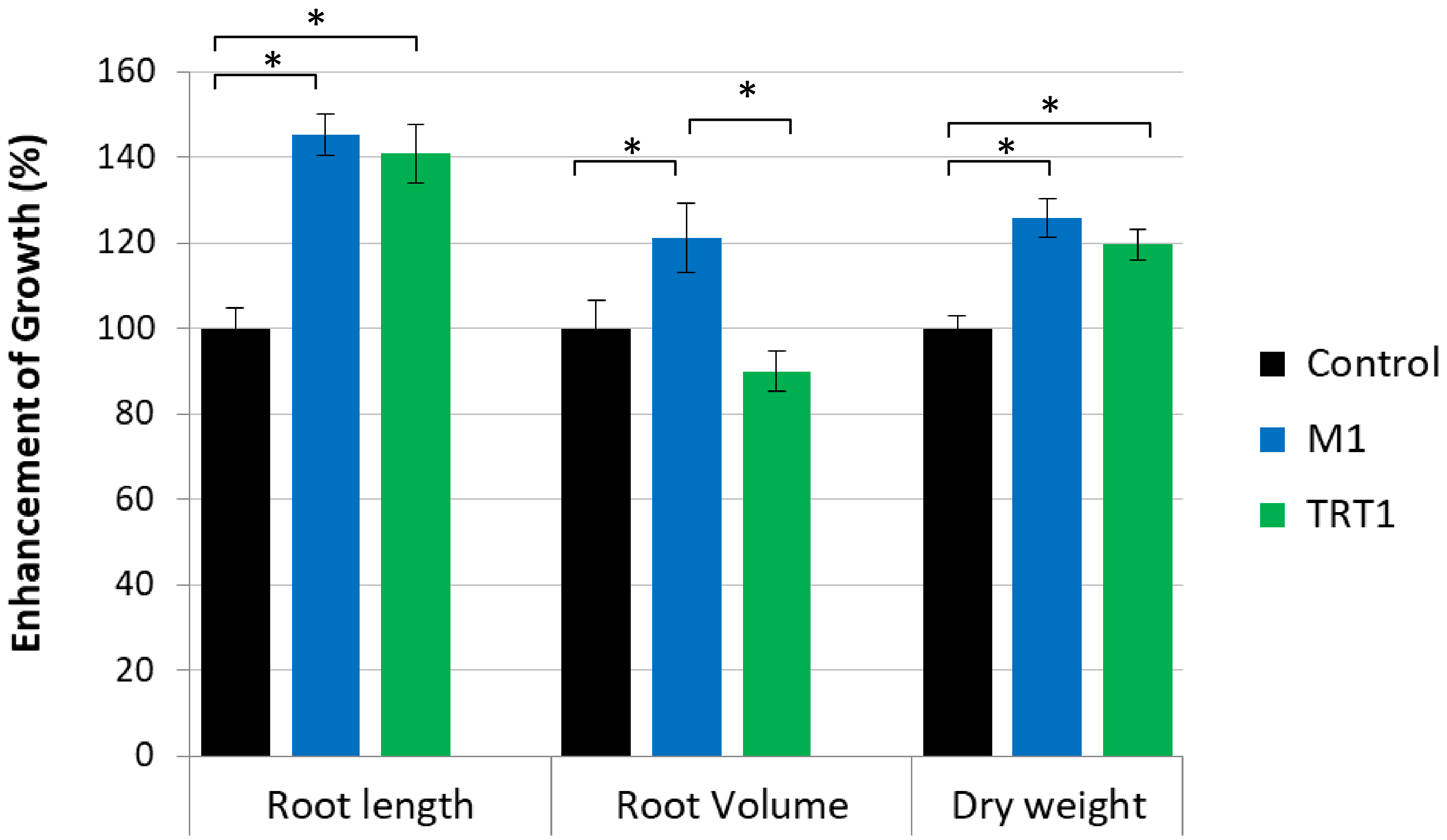

Fig. S7 - Plant growth promotion assay in maize 7 days after inoculation with strains M1 (wild-type) and TRT1. Maize seedlings were inoculated with bacterial suspensions containing 10^5 bacteria.mL^{-1}, and parameters were measured after 7 days. For each treatment was measured at least 30 plants. Asterisks indicate statistically significant difference at $p \leq 0.05$ (*) (t test, Assistat program).
